# How to bring research evidence into policy? Synthesizing strategies of five research projects in low-and middle-income countries

**DOI:** 10.1186/s12961-020-00646-1

**Published:** 2021-03-06

**Authors:** Séverine Erismann, Maria Amalia Pesantes, David Beran, Andrea Leuenberger, Andrea Farnham, Monica Berger Gonzalez de White, Niklaus Daniel Labhardt, Fabrizio Tediosi, Patricia Akweongo, August Kuwawenaruwa, Jakob Zinsstag, Fritz Brugger, Claire Somerville, Kaspar Wyss, Helen Prytherch

**Affiliations:** 1 Swiss Tropical and Public Health Institute, Basel, Switzerland; 2 University of Basel, Basel, Switzerland; 3 CRONICAS Centre of Excellence in Chronic Diseases, Universidad Peruana Cayetano Heredia, Lima, Peru; 4 Division of Tropical and Humanitarian Medicine, University of Geneva and Geneva University Hospitals, Geneva, Switzerland; 5 Centro de Estudios en Salud, Universidad del Valle de Guatemala, Guatemala, Guatemala; 6 Department of Infectious Diseases and Hospital Epidemiology, University Hospital Basel, Basel, Switzerland; 7 School of Public Health, College of Health Sciences, University of Ghana, Accra, Ghana; 8 Ifakara Health Institute, Plot 463, Kiko Avenue Mikocheni, Dar es Salaam, Tanzania; 9 Swiss Federal Institute of Technology Zurich, Zurich, Switzerland; 10 Gender Centre, Graduate Institute of International and Development Studies, Geneva, Switzerland

**Keywords:** Evidence-based policy-making, Research for development

## Abstract

**Background:**

Addressing the uptake of research findings into policy-making is increasingly important for researchers who ultimately seek to contribute to improved health outcomes. The aims of the Swiss Programme for Research on Global Issues for Development (r4d Programme) initiated by the Swiss National Science Foundation and the Swiss Agency for Development and Cooperation are to create and disseminate knowledge that supports policy changes in the context of the 2030 Agenda for Sustainable Development. This paper reports on five r4d research projects and shows how researchers engage with various stakeholders, including policy-makers, in order to assure uptake of the research results.

**Methods:**

Eleven in-depth interviews were conducted with principal investigators and their research partners from five r4d projects, using a semi-structured interview guide. The interviews explored the process of how stakeholders and policy-makers were engaged in the research project.

**Results:**

Three key strategies were identified as fostering research uptake into policies and practices: (S1) stakeholders directly engaged with and sought evidence from researchers; (S2) stakeholders were involved in the design and throughout the implementation of the research project; and (S3) stakeholders engaged in participatory and transdisciplinary research approaches to coproduce knowledge and inform policy. In the first strategy, research evidence was directly taken up by international stakeholders as they were actively seeking new evidence on a very specific topic to up-date international guidelines. In the second strategy, examples from two r4d projects show that collaboration with stakeholders from early on in the projects increased the likelihood of translating research into policy, but that the latter was more effective in a supportive and stable policy environment. The third strategy adopted by two other r4d projects demonstrates the benefits of promoting colearning as a way to address potential power dynamics and working effectively across the local policy landscape through robust research partnerships.

**Conclusions:**

This paper provides insights into the different strategies that facilitate collaboration and communication between stakeholders, including policy-makers, and researchers. However, it remains necessary to increase our understanding of the interests and motivations of the different actors involved in the process of influencing policy, identify clear policy-influencing objectives and provide more institutional support to engage in this complex and time-intensive process.

## Background

Increasingly, research funders are asking their grantees to address the uptake of research findings into decision-making processes and policy-making [1, 2]. This growing trend is a response to a need for real-world and context-sensitive evidence to respond to and address complex health systems and health service delivery bottlenecks faced by policy-makers, health practitioners, communities and other actors that require more than single interventions to induce large-scale change [3]. Moreover, there is growing pressure for applied and implementation research to be relevant, demonstrate value for money and result in high-impact publications. The relevance of ensuring the translation of research into practice is also reflected in growing support for research projects with concrete requirements regarding the evaluation of their impact of science on society [4].

One example of the above is the Swiss Programme for Research on Global Issues for Development (r4d Programme) initiated by the Swiss National Science Foundation (SNSF) and the Swiss Agency for Development and Cooperation (SDC) covering the period 2012–2022. The r4d Programme is aimed at researchers in Switzerland and low-and middle-income countries (LMICs) conducting projects that specifically focus on poverty reduction and the protection of public goods in developing countries. Its specific objectives are to create and disseminate knowledge that supports policy-making in the area of global development and foster research on global issues in the context of the 2030 Agenda for Sustainable Development [5, 6].

While the linkage of research to policy is strongly encouraged by research funding agencies, the uptake of research evidence by policy-makers to establish new laws and regulations or to improve policies to solve a problem or enhance implementation effectiveness, especially in LMICs, remains weak [2, 7]. This is often referred to as the gap between research and policy [8]. One of the factors that was identified with the dearth of research uptake in previous studies is a lack of evidence that is context sensitive, timely and relevant for policy-makers; other factors include difficulties in accessing existing evidence, challenges with correctly interpreting and using existing evidence [7, 9] and also a lack of interest from policy-makers in the use and uptake of evidence [10]. Using the SNSF r4d funding scheme, our aim is to show how researchers have engaged with stakeholders, including policy-makers, from the onset of a research project, in order to identify strategies for evidence uptake and use.

As part of the r4d Programme, several synthesis initiatives have been launched to disseminate the research evidence from the r4d projects and increase its impact (http://www.r4d.ch/r4d programme/synthesis). The aim of one of these synthesis initiatives is to support knowledge translation and exchange, as well as knowledge diffusion and dissemination among 15 r4d projects focusing on public health. More specifically, the aim is to facilitate the uptake of findings for the benefit of societies in LMICs, especially with regards to social inclusion and gender equity in the drive towards universal health coverage (UHC) and the 2030 Agenda for Sustainable Development [6]. The present study and resulting article are part of this synthesis initiative.

In this article, we present—through five case studies—strategies to translate and bridge evidence emerging from research into policy-making and decision-making. We rely on the experiences of five public health projects within the r4d research initiative. This paper describes these experiences, reports on the lessons learnt and outlines important features and challenges of engaging in this process using the researchers’ perspectives. This paper contributes to the body of literature on research translation by highlighting concrete examples and successful strategies for the uptake of research evidence in policy formulation.

## Methods

Invitations were sent out to researchers working on projects within the r4d Programme to share their experiences with the project. Based on the interest shown by researchers, five projects were selected by the authors to demonstrate the different approaches and strategies used in the r4d projects with the aim to influence policy. Researchers were asked to share descriptions of the different approaches used in seeking to influence the uptake of research results by policy-makers. Each project represents a case study with emphasis on the main features of their translational approaches and the challenges, enablers and successes encountered.

The different research–policy engagement strategies were identified through data analysis of the interviews conducted within the framework of the five r4d case studies and were inspired by the work conducted by Uzochukwu and colleagues in Nigeria [2], who described four detailed strategies to support evidence-informed policy-making: (1) policy-makers and stakeholders seeking evidence from researchers; (2) involving stakeholders in designing objectives of a research project and throughout the research period; (3) facilitating policy-maker–researcher engagement in optimizing ways of using research findings to influence policy and practice; (4) active dissemination of own research findings to relevant stakeholders and policy-makers (see Table 1).

**Table 1:** Framework strategies by Uzochukwu et al. [2] and strategies identified in the synthesis work

Framework provided by Uzochukwu et al. [2]	Description of the strategy by Uzochuwku et al. [2]	Strategies identified in the r4d synthesis work	Differences encountered
S1: Policy-makers and stakeholders seeking evidence from researchers	Stakeholders request evidence on implementation/scaling up. The research is either funded by policy-makers and decision-makers or by an external agency. Thus, the strategy is about being responsive to opportunities created by policy-makers calling for evidence	S1: Stakeholders directly engaged with and sought evidence from researchers	In the research projects investigated, the funding scheme provided by the SNSF is common to all. Stakeholders, including policy-makers actively sought evidence from researchers, but did not initiate empirical research studies as suggested in the framework provided by Uzochuwku et al. [2]
S2: Involving stakeholders in designing objectives of a research project and throughout the research period	Stakeholders were involved in the designing of the research and throughout the project period. The research questions and objectives arose from these experiences of the MoH and involved stakeholders, such as the NHIS	S2: Stakeholders were involved in the design and throughout the implementation of the research project	According to S2 as described by Uzochukwu et al. [2], policy-makers initiated empirical research studies. Similarly, we found stakeholders involved in the design and implementation of r4d research projects from early on, but it was not the policy-makers themselves initiating the research studies
S3: Facilitating policy-maker–researcher engagement in optimizing ways of using research findings to influence policy and practice	This strategy focused on developing policy-maker capacity to use research evidence for policy-making by, for example, enhancing institutional capacity among senior and middle level health managers within the MoH to use research evidence to influence policy-making	–	Among the participating r4d projects, we did not directly encounter this strategy of enhancing policy-makers’ capacity to use research evidence. However, in all the five r4d case studies studied, and hence as part of the three identified strategies, initiatives for strengthening research capacity were undertaken to train national researchers and strengthen the association with intstitutions directly involved
S4: Active dissemination of own research findings to relevant stakeholders and policy-makers	They key concepts of knowledge transfer were employed including research reports, peer reviewed papers, conference presentations and policy briefs	S3: Stakeholders engaged in participatory and transdisciplinary research approaches to co-produce knowledge and inform policy	According to S4 by Uzochukwu et al. [2], researchers in researcher-initiated empirical research studies actively disseminate their own findings We did not encounter this strategy as such among the r4d case studies, as all of the participating resarchers were active in disseminating their research findings. Instead, our results showed that co-creation and transdisciplinary research approaches came close to this active knowledge transfer and even wents beyond dissemination to knowledge co-production by a range of stakeholders, which is why we have further elaborated this strategy

*MoH* Ministry of Health,* NHIS* National Health Insurance Scheme,* SNSF* Swiss National Science Foundation

In using the term stakeholder, we apply the following definition by Brinkerhoff and Crosby [11]: “A stakeholder is an individual or group that makes a difference or that can affect or be affected by the achievement of the organization’s objectives”. Hence, individual stakeholders can include politicians (heads of state and legislators), government bureaucrats and technocrats from various sectors (e.g. health), but also representatives of civil society organizations and support groups [12].

### Data collection

Eleven in-depth interviews with principal investigators and their research partners from five r4d projects were conducted by the first author, using a semi-structured interview guide. The interview guide covered the following themes: (1) How were stakeholders involved in the research project? (2) Was there uptake of research evidence in national/international policies? (3) How were research results disseminated? (4) What were the challenges or obstacles encountered in disseminating and translating evidence from research to policy? The interview duration was between 30 and 45 min. Seven interviews were conducted with researchers based in Switzerland and four with researchers in LMICs. At least two interviews were conducted for each r4d case study.

### Data management and analysis

Of the 11 interviews, nine were audio recorded and notes taken. Audio files were transcribed verbatim by the same researcher. Two interviews were not recorded, but detailed notes were taken during the interview.

A qualitative content analysis method was used in order to organize and structure both the manifest and latent content [13]. Aligned to overall study questions, essential content was identified by the first author, which involved a process of generating a provisional list of themes of interest that were based on the study objectives, including stakeholder involvement in the generation of research questions, research process, generation of results and dissemination of research findings, as well as challenges to research dissemination and policy uptake. In a next step, the transcripts were sorted and grouped by the first author according to the coding scheme for analysis. This involved using the content summary analysis method, which consists of reducing the textual content and preserving only the essential content in order to produce a short text [14]. As several co-authors were interviewed, they validated that their perspective was not misinterpreted or misrepresented.

## Results

Three key strategies were identified for research uptake into policy and practice throughout the data collection of this synthesis initiative: (S1) stakeholders directly engaged with and sought evidence from researchers; (S2) stakeholders were involved in the design and throughout the implementation of the research project; and (S3) stakeholders engaged in participatory and transdisciplinary research approaches to co-produce knowledge and inform policy. The first two strategies (S1, S2) are in line with Uzochukwu and colleagues’ work [2], and the third strategy (S3) is an additional category based on the experiences of researchers in r4d projects [2]. Each r4d project is described in more detail as a case study in one of these three strategies (Table 2).

**Table 2:** R4d Programme case studies and strategies identified in the synthesis work

Project name	Geographic location	Strategies identified in the synthesis work	Type of r4d projects’ policy engagement according to the strategies identified	Website
Improving the HIV care cascade in Lesotho: towards 90-90-90—a research collaboration with the MoH	Lesotho	S1: stakeholders directly engaged with and sought evidence from researchers	International stakeholders contacted the r4d research team based on the publication of the research protocol and directly took up published research findings. As a result, same-day ART initiation was newly included in WHO guidelines. National stakeholders were also involved from the very beginning in the project design	http://www.r4d.ch/modules/thematically-open-research/hiv-care-cascade
Health systems governance for an inclusive and sustainable social health protection in Ghana and Tanzania	Ghana, Tanzania	S2: stakeholders were involved in the design and throughout the implementation of the research project	National stakeholders involved since the beginning through an advisory function in country advisory boards. Participation in national workshops to identify research objectives and design the research project As a result of the evidence produced, policy documents were up-dated to improve identification of the poor to increase their enrollment, and tariffs were adjusted to reduce optional payment plan by clients	http://www.r4d.ch/modules/public-health/health-systems-governance
Health impact assessment for engaging natural resource extraction projects in sustainable development in producer regions (HIA4SD)	Burkina Faso, Ghana, Mozambique, Tanzania		National stakeholders involved since the beginning through kick-off meetings—mining is a highly politicized environment in most countries). Evidence is still being produced and dissemination is part of second phase (starting in 2021)	http://www.r4d.ch/modules/public-health/health-impact-assessment
Intercultural transdisciplinary in Guatemala and Peru: a North-South–South learning platform for culturally pertinent public health provision systems for indigenous populations	Guatemala	S3: stakeholders engaged in participatory and transdisciplinary research approaches to co-produce knowledge and inform policy	Co-creative, participatory research approaches with involvement of stakeholders in designing objectives of the research and throughout project implementation Each country had also a local advisory board made up with people in decision-making positions to plan research activities before project onset and to discuss ongoing research activities	http://www.r4d.ch/modules/thematically-open-research/culturally-pertinent-health-provision
Addressing the double burden of disease: Improving health systems for non-communicable and neglected tropical diseases	Nepal, Mozambique, Peru			http://www.r4d.ch/modules/public-health/addressing-double-burden-disease

*ART* Antiretroviral therapy,* HIA4SD* health impact assessment for engaging natural resource extraction projects in sustainable development in producer regions

### S1: stakeholders directly engaged with and sought evidence from researchers

In this strategy, international stakeholders requested evidence from the research team. This is a unique (and rare) strategy, as stated by Uzochukwu et al. [2], and often involves a policy window of opportunity in which stakeholders, including policy-makers, are looking to solve a particular problem, which coincides with the publishing of a scientific report or paper and the interests of these same groups [15, 16].

#### Improving the HIV care cascade in Lesotho: towards 90-90-90—a research collaboration with the Ministry of Health of Lesotho

In this r4d project, the research team was approached by the International Aids Society (IAS) and the World Health Organization (WHO) in Geneva, based on the publication of their study protocol [17], introducing their innovative research approach of same-day antiretroviral therapy (ART) initiation in rural communities in Lesotho:

“They [international stakeholders] were all keen of getting the results out and requested evidence of the randomized controlled trials. We shared the results confidentially with WHO as soon as we had the data and thereafter published the results in a journal with a wide reach. WHO as well as other international guidelines and policy committees took up the recommendation of same-day ART initiation and informed global guidelines” (Researcher 1).

As a result, many HIV programmes in sub-Saharan Africa as well as in the global north have adopted the practice of offering rapid-start ART to persons who test HIV positive even outside a health facility. In this example, the policy window and direct stakeholder engagement was crucial for the effective translation and uptake of research evidence.

Furthermore, by closely collaborating with national policy-makers, the research team advocated for the setting up of a research database and of knowledge management units within the Ministry of Health (MoH) of Lesotho, which have been successfully established. The members of the research project consortia have also initiated a national research symposium on a bi-annual basis, which is chaired by the MoH with the aim of facilitating the dissemination and uptake of research findings.

### S2: Stakeholders were involved in the design and throughout the implementation of the research project

In this strategy, policy uptake is facilitated through stakeholder engagement from the beginning as well as during the conduct of research activities, through participating at workshops or functioning in the governance of the projects. Two r4d projects illustrate this strategy.

#### Health system governance for an inclusive and sustainable social health protection in Ghana and Tanzania

This project established a Country Advisory Group (CAG) at the start that included representatives of the main stakeholders of the social health protection systems. The CAGs were involved in all phases of the project, from the definition of the research plans to the dissemination of the results. The specific research questions addressed by the project emerged from the interactions with these main stakeholders, i.e. national policy-makers, healthcare providers and members of the social health protection schemes (the NHIS and the Livelihood Empowerment Against Poverty schemes in Ghana; and the National Health Insurance Fund, the Community Health Funds and the Tanzania Social Action Fund in Tanzania). Specifically in Ghana, the following stakeholders played a major role in shaping the research plan: the Ministry of Gender Children and Social Protection (MGCSP), the Ghana Health Service (Policy Planning and Monitoring and Evaluation Division; Research and Development Division), the National Health Insurance Authority (NHIA) and the Associations of Private Health Care Providers. In Tanzania, a major role was played by the Ministry of Health, Community, Development, Gender, Elderly and Children, the President’s Office—Regional Administration and Local Government, by representatives of civil society organizations, such as Sikika, by the SDC (Swiss Agency for Development and Cooperation) Health Promotion and System Strengthening project and by the SDC-supported development programme.

These stakeholders were subsequently involved in steering the research, as captured by a researcher:

“In Ghana, it was a balanced relationship. They were involved since the very beginning of the project in articulating what the information gap at policy level is, formulating the research questions and understanding the methods/what is feasible. In Tanzania, where the policy landscape is more fragmented, it was very important to listen to the voices of several different stakeholders” (Researcher 2).

The stakeholder consultations in Ghana and Tanzania initially involved discussions on the relevance of the research plans to address the existing gaps in strengthening the social health protection scheme, the synergies with other research initiatives and the feasibility of implementing the proposed research. Later on in the project, the consultation process involved reviewing and discussing the focus of the research and the appropriateness of the research aims in light of decisions and reforms that were under discussion by the government but not in the public domain. This led to revision of the research questions as they would have become redundant when such reforms were made public, especially in Ghana. These consultation processes were more formal in Ghana and more informal in Tanzania, but they were very informative and had a tangible impact on the research plans, which were revised according to the feedback received. However, the research teams were always independent in deciding on the research methodology and in interpreting the results. The in-country dissemination of the results at the end of the first phase of the project informed the decisions to be made on the research plan for the second phase and provided the opportunity to discuss policy implications based on the results of the first phase. Because of this close collaboration and engagement with stakeholders, the results of the studies were widely disseminated in Ghana. Two of the main findings of the project were particularly considered by these stakeholders. According to the researcher:

“First, the study results showed that even though people registered with the NHIS they continued to pay out of pocket for health services. The reasons for this were delays in reimbursement by NHIS, escalating prices of drugs and medical products, low tariffs, lack of trust between providers and NHIA and inefficiencies. Secondly, the results showed that the current system of targeting the poor is not working properly, with more than half of people registered in the NHIS as indigents being in the non-poor socio-economic groups. These results contributed to inform decisions regarding the revision of the NHIA reimbursement tariffs, and to improve the identification of the poor to be exempted from paying the NHIS premium, in collaboration with the MGCSP” (Researcher 3).

In Tanzania, research was conducted to assess the effects of the public private partnership, referred as the Jazia Prime Vendor System (Jazia PVS), on improving access to medicines in the Dodoma and Morogoro regions in Tanzania. This is one of the reforms in the area of supply chain management taking place in the country. Results showed that a number of accountability mechanisms (inventory and financial auditing, close monitoring of standard operating procedures) implemented in conjunction with Jazia PVS contributed positively to the performance of Jazia PVS. Participants’ acceptability of Jazia PVS was influenced by the increased availability of essential medicines at the facilities, higher-order fulfilment rates and timely delivery of the consignment [18–20].

The findings from this study were disseminated during the national meeting attended by various stakeholders, including CAG members, government officials and policy-makers. In addition, the findings were used to inform the national scale-up of the Jazia PVS intervention as the government of Tanzania decided to scale up the Jazia PVS to all the 23 regions in 2018. Moreover, the results/manuscripts were published or submitted to peer-reviewed journals [18–20], enabling other countries intending to adopt such innovate public–private partnerships for improvement of the in-country pharmaceutical supply chain to learn from Jazia PVS in Tanzania.

#### Health impact assessment for engaging natural resource extraction projects in sustainable development in producer regions (HIA4SD)

In this r4d project, stakeholders were involved from the outset through their participation in the project launch meeting and in regular consortium meetings. The project is a collaboration between the Swiss Tropical and Public Health Institute (Swiss TPH), the Center for Development and Cooperation (NADEL) at the Swiss Federal Institute of Technology in Zurich/Switzerland and national research institutes, namely the Institut de Recherches en Sciences de la Santé in Burkina Faso, the University of Health and Allied Sciences in Ghana, the Centro de Investigação em Saúde de Manhiça in Mozambique and the Ifakara Health Institute in Tanzania [21]. The involvement of key stakeholders from the government, civil society, private sector and research community in an engaged dialogue from the beginning iss of central importance in this project, as in most cases mining is a highly politicized topic. To promote the immediate integration of research findings into policy, the project is organized into two streams, namely an “impact research stream” and a “governance stream”, that work in parallel. While the impact research stream is focused on evidence generation to support the uptake of health impact assessment (HIA) in Africa, the governance stream is focused on understanding the policy terrain and consequently the pathways that need to be utilized to support translation of the evidence into policy and practice. The second phase of the study is devoted to the dissemination of research findings into policy at the national and local levels, including capacity-building activities for national stakeholders. As the HIA4SD project examines operational questions of relevance for guiding both policy-making and decision-making, team members sought to regularly engage with and inform the national stakeholders. According to the researcher:

“Strategies employed to influence policy vary according to the country, but included regular stakeholder workshops, participation in a new national platform launched to discuss issues around mining in Mozambique, development of policy briefs, strengthened collaborations with national ministries of health, discussion of results and advocacy with policy makers, and conference presentation of findings” (Researcher 4).

In these two case examples, continuous stakeholder engagement was considered essential to translate and disseminate research evidence. Thus, beyond the stage of setting the objectives, contact with stakeholders was active and maintained on a regular basis through regular exchanges with stakeholder groups during workshops or meetings, which facilitated the dissemination and uptake of the research results. While the time and level of meaningful interaction varied across the countries and workshops, all meetings were well attended by participants from varied levels of government, MoHs, nongovernmental organizations and private industry, prompting spirited discussion and insight from these groups. All stakeholders were willing to attend these workshops as part of the scope of their professional duties.

### S3: stakeholders engaged in participatory and transdisciplinary research approaches to co-produce knowledge and inform policy

In the two examples presented in this section, the research questions and approaches arose through community and stakeholder participation in the research and intervention design itself. The methodology adopted allowed them to engage, design research, act, share and sustain partnerships between the communities, the involved stakeholders and researchers [22]. These participatory research approaches facilitated grassroot-level policy and practice changes which were not researcher nor policy maker led, and that show promising approaches for developing culturally aligned solutions [23]. Policy makers at both the regional and national levels were invited to be part of the participatory research approach: they were interviewed during the initial stage, then the research results were presented and discussed with them; thereafter, we had several meetings to co-create potential interventions to address the identified problems, with the aim to directly engage in the research and intervention design itself in partnerships with the community stakeholders, including local leaders, and the researchers.

#### Surveillance and response to zoonotic diseases in Maya communities of Guatemala: a case for OneHealth

The research was embedded in a collaboration between the Universidad del Valle in Guatemala, the MoH, the Ministry of Animal Production and Health, the Maya Qéqchi’ Council of Elders, TIGO Telecommunications Foundation and the community development councils. The objective of this r4d programme was to set up integrated animal–human disease surveillance (OneHealth) in Maya communities in Guatemala. The research approach arose from a context of medical pluralism, where communities have access to and use two different medical systems: (1) the modern Western medical system and (2) traditional Maya medicine [24].

Researchers and community members collaborated at all stages of the research process, including the planning stage. Even before the grant proposal was finalized, researchers met with the communities that, should the funding come through, would be invited to participate in the research. According to the researchers:

“The project was set up through a transdisciplinary process, with academic and non-academic actors—including national, local and traditional authorities—involved in the problem through a collaborative design, analysis, dissemination and research translation. It was a co-producing transformative process—transferring knowledge between academic and non-academic stakeholders in plenary sessions and through group work. These meetings were held every year to continuously follow up the progress of the process” (Researcher 7).

The active engagement and collaboration by the community and stakeholders facilitated the acceptability of the study results and hence its dissemination, captured by the researchers as follows:

“The main result was that they allowed a frank discussion between Maya medical exponents in human–animal health and Western medicine, which allowed the patients and the animal holders to avoid the cognitive dissonance and so that the patients or the animal holders can choose freely what they want. Cognitive dissonance exists if one system dominates the other—or refutes the other” (Researcher 7).

“After all stakeholders discussed the research evidence produced jointly, an unprecedented process of collaboration between Government authorities and communities followed to develop three joint responses: a) education campaigns led by local teachers in tandem with the Ministry of Education, b) communication strategies at regional levels led by the Human and Animal Health authorities along with traditional Maya Ajilonel (medicine specialists), and c) a policy framework for producing a OneHealth approach led by Central Government authorities” (Researcher 8).

The process of mutual learning throughout the project produced a new level of awareness, facilitating culturally pertinent and socially robust responses that overcame a historical tendency of unilateral policy making based solely on Western values and preferences. As the project implemented a new approach to monitoring animal and human populations, the involvement of regional teams from the different ministries (Health, Livestock and Agriculture) throughout all the phases of methodological design, data collection, posterior data analysis and design of specific interventions for the local population (transformation of scientific results into actions for public health improvement) was essential to ensuring that the approach used secured the regional authorities’ commitment to defining new policies for immediate application in their territory. Accordingly, this also contributed towards the development of a OneHealth national strategy for Guatemala in which ministries start to cooperate to take up priority issues.

#### Addressing the double burden of disease: improving health systems for non-communicable and neglected tropical diseases (Community Health System Innovation [COHESION])

Together with three Swiss academic partners, this r4d project examined the challenges of a double burden of non-communicable and neglected tropical diseases at the primary healthcare level in vulnerable populations in Mozambique, Nepal and Peru. Community participation and co-creation were key elements of the project’s approach. The work conducted in Peru illustrates this approach:

“At the beginning, the people who were involved were respondents, but then they became active participants. So it was this active engagement and the changing of roles, giving feedback not just from the research responses but also from being involved in the process, which helped to design and create interventions together with the research team” (Researcher 5).

This participatory approach to co-creation actively sought a diverse range of stakeholders, including community members, primary healthcare workers, and regional and national health authorities. The co-creation approach to participatory research enables context-specific variation in methodological design, a critical element when studying three very different countries and health systems. Central to all aspects was a feedback loop whereby early findings were shared with research participants for further elaboration and iteration.

As active co-creators of the research process, local communities developed high levels of trust in the methodology and data, with the result that researchers achieved deeper “buy-in” which in turn is known to enhance the uptake of findings by decision-makers [25] as communities in which research is being undertaken play a central role in the decision-making process [26].

### Challenges to research uptake in health policy identified by r4d researchers

During the interviews, r4d researchers identified several challenges to research utilization and uptake into policy. These challenges are summarized and highlighted in Table 3.

**Table 3:** Challenges to research uptake in health policy identified by r4d researchers

Challenge	Relevant quote
1. Time investment, translation of research findings and the role of researchers	“Is it the role and responsibility of researchers? Do they have the skills and time to do it? It takes a long time to synthesize multiple papers in one page” (Researcher 5) “It is difficult as it takes longer to do this translation. It is positive to engage with policy makers in a research project, but it takes much more work than what you would think in the beginning. Sometimes it is challenging to engage with policy makers—research outputs take a long time; plus you may not have as many research results as you would like to” (Researcher 2)
2. Problem of scale and objectivity	“You have to be very cautious in presenting findings to policy or decision-makers when they intend to use them. Findings in one small study do not necessarily make for generalities. One has to be very careful in translating findings to policy without first systematically reviewing a whole body of evidence. (Researcher 6) “What appears to be relevant in Peru might be very different to what is relevant in Nepal. It would be unethical to try to draw some similarities between them, or suggest policy changes on the basis of findings at these scales” (Researcher 6)
3. Frequent staff change at governmental level	“There has been a lot of change in the team of the national health insurance authority; this makes it difficult to see how they are using the information” (Researcher 3)
4. Diverging interests and timelines	“There is often a disconnect between data/science, research funders and policy makers interest” (Researcher 2)

## Discussion

Three key strategies identified for research uptake in policy and practice are described in this paper, namely: (S1) stakeholders directly engaged with and sought evidence from researchers; (S2) stakeholders were involved in the design and throughout the implementation of the research project; and (S3) stakeholders engaged in participatory and transdisciplinary research approaches to co-produce knowledge and inform policy. These strategies are in line with the overall objectives of the r4d projects, which are to generate scientific knowledge and research-based solutions to reduce poverty and global risks in LMICs, and also to offer national and international stakeholders integrated approaches to solving problems [5]. In the course of our synthesis work, we found that several lessons could be learned from the three strategies identified for research uptake in policy and practice.

### S1: raising awareness of planned research to attract stakeholder involvement

The actual uptake of research findings in policy was most direct in the case of the first strategy (S1), in which IAS and WHO stakeholders were wanting new knowledge on HIV and same-day ART initiation, and were actively seeking new evidence on these specific topics. The findings published in peer-reviewed journals were then taken up by these stakeholders to update international policies and guidelines on rapid ART initiation [27]. This was also found in other studies, highlighting the importance of the timeliness and relevance of findings and the production of credible and trustworthy reports, among others, as key factors in promoting the use of research evidence in policy [2, 28].

### S2: sustainable collaborations in a supportive policy environment with stakeholder engagement from early on and throughout the research process

With regards to the second strategy (S2), we found that constant collaboration with an advisory and steering group composed of diverse stakeholders, including policy-makers, from early on promotes the uptake and use of research evidence. In line with findings from other studies [2], the experiences encountered in the r4d public health projects show that early involvement of stakeholders in the processes to identify the research problem and set the priorities facilitated the continuous exchange of information that might ultimately influence policy. The r4d project on social governance mechanisms in Ghana highlight that the evidence produced influenced policy documents (identification of the poor and tariff adjustments), but that frequent changes government officials made it difficult to maintain a close relationship between the researchers and the governmental agencies/policy stakeholders. From this, we draw the conclusion that research approaches need to be more adaptive and flexible to be successful in an unsupportive or unstable policy environment to ensure continuity in promoting the dissemination and uptake of research evidence in policy-making. One possible manner to secure this transformation is for researchers to apply for additional funding after the grant is finished. Other studies have also come to this conclusion, thereby demonstrating the key role of a supportive and effective policy environment that includes some degree of independence in governance and financing, strong links to stakeholders that facilitate trust and influence and also the capacity within the government workforce to process and apply policy advice developed by the research findings [29]. By involving stakeholders in the process of identifying research objectives and designing the project, as seen particularly in the r4d case studies on social health protection in Ghana and Tanzania and the HI4SD, but also in the HIV care cascade in Lesotho, the research approach responded to the need of locally led and demand-driven research in these countries, strengthening local research capacities and institutions, but also investing in research that is aligned with the national research priorities. As highlighted by other authors, advantages of this “demand-driven” approach is that it tailors research questions to local needs, helps to strengthen local individual and organizational capacities and provides a realized stringent framework on which a research project should deliver outcomes [30, 31].

### S3: co-creation and equal partnerships

The third strategy with a strong participatory approach, such as that adopted by two r4d projects, OneHealth in Guatemala and COHESION, demonstrates benefits to promoting co-learning as a way to minimize the impact of unequal power dynamics and to work effectively across the local policy landscape through equal partnerships. It also facilitates identifying solutions that are culturally pertinent, socially more robust and implementable.

The approaches of co-creation, equal participation and stakeholder involvement used in the research projects raise questions of ‘governance’, that is the way rules, norms and actions are structured, sustained and regulated by public and para-public actors to condition the engagement and impact of public involvement activities [32, 33]. Through stakeholder involvement in setting the agenda and designing the research projects, as shown in the case studies on social protection in Ghana and Tanzania and the HI4SD project, but particularly in the two projects using a co-creation approach, the engagement of a range of stakeholders serves to make the health research systems a participaant in the endeavor that then has the capacity to promote changes in the healthcare system it aims to serve. By establishing a shared vision with a public involvement agenda and through the collaborative efforts of various stakeholders, as we found particularly in the co-creation approach, supportive health research systems are established. This leads to greater public advancement through collaborative actions, thereby tackling the stated problems of the health systems [34].

## Challenges

There were four key challenges mentioned by the respondents during the interviews to research uptake in policy making. The first was the necessary time investment by researchers to translate the result and develop policy advocacy products for the different audiences. This challenge is all the more difficult because research evidence and tangible products only become available towards the end of a research project, leaving only a short window of opportunity for exchange and engagement. There seems to be a need for wider discussion on the role of researchers in influencing policy. The concerns raised included whether influencing policy is actually a role for researchers and whether researchers have the right skills to be effective in persuasion or network formation [35]. Conversely, researchers may be in a good position to engage in the policy process if they enjoy finding solutions to complex problems while working with diverse and collaborative groups in partnerships [36, 37]. The rationale for engaging in such a process needs to be clarified in advance: is the aim to frame an existing problem, or is it to simply measure the issues at stake and provide sound evidence according to an existing frame? Regarding the the former, how far should researchers go to be useful and influential in the policy process or to present challenges faced by vulnerable populations [37]? While fully engaging in the policy process may be the best approach for researchers to achieve credibility and impact, there may also be significant consequences, such as the risk of political interests undermining the methodological rigour of academic research (being considered as academic ‘lightweight’ among one’s peer group) [38–41]. For researchers there is also considerable opportunity costs because engaging in the policy-influencing process is a time-consuming activity [35], with no clear guarantee of the impact of success [37]. It is therefore crucial to consider the investment and overall time researchers may have to spend to engage [35], and how this time and investment can be distributed between actual research and the production of outreach products, such as policy briefs, presentation of research findings as policy narratives [35] and the setting-up of alliances, building of networks and exploitation of windows of opportunity for policy change [37].

The second challenge included the issue of scale and objectivity, as most of the projects are not scaled or national-level studies and thus are highly context specific. The difficulty to measure the contributions of a single research project or study in terms of policy outcomes was also highlighted, particularly in view of the different understandings among researchers and funders on the possible policy impacts of the research, which can range from guiding policy-makers to understand a situation or problem (awareness raising) to influencing a particular course of action by establishing new or revising existing policies. This has also been emphasized in the Evidence Peter Principle [42], showing that single studies are often inappropriately used to make global policy statements for which they are not suitable. To make global policy statements, an assessment of the global evidence in systematic reviews is needed [42, 43].

The third challenge mentioned was the frequent changes in staff at the governmental level, which demanded continuous interactions between r4d researchers and stakeholders, highlighting the need for more adaptive and flexible research approaches. These should include a thorough analytical process prior to implementation in historical, sociopolitical and economic aspects, power differentials and context; backward planning exercises to check assumptions; and conflict transformation and negotiation skills in order to be able to constantly adapt to changing contexts. In line with our research findings, when researchers make the time investment needed to engage in the policy-influencing process, an opportunity is provided to getting know the involved stakeholders better and improve their understanding of the policy world in practice, but also to build diverse and longer-term networks [37, 44] and to identify policy problems and the appropriate stakeholders to work with [45, 46]. Engaging a diverse range of stakeholders through co-designing the research is widely held to be practically the best way to guarantee the uptake and use of evidence in policy through a more dynamic research approach [47]. However, the development of networks and contacts for collaboration, as well as the skills to do so, takes time and effort and is an ongoing process [48], factors which need to be acknowledged more widely.

Lastly, the fourth challenge related to research uptake was the diverging interests between researchers, research funding bodies and stakeholders. Time was identified as a limiting factor from the perspective of the design of the research project. Most research projects, including the r4d projects, are funded for 3–4 years [5]. It takes a considerable amount of time to generate new research results, and often these are more likely to be produced for further use at the end of a project. If researchers should engage more fully in the policy process to secure meaningful impact, it is critical to discuss the extent to which they have the skills, resources and institutional support to do so [37], as well as how projects could be set up differently. This could be done either by the funders in providing the necessary support that allows researchers to have the means to impact policy, or by the researchers in the design of their project to take on board the different strategies to influence evidence use and uptake. In moving forward, defining shared goals from the outset between funders and the researchers might translate to more achievable milestones in terms of which policy issue, theme or process a research project aims to change in order to effectively influence policy [49]. This would help to identify the resources and budget needed by the funders in order for the researchers to engage with more resources over a longer time span in this process.

## Limitations

Interviews were limited to researchers of the r4d projects and did not include local stakeholders. Therefore, the synthesis work, including the analysis and results, reflects solely the perspective of researchers. We are aware that had we included a range of stakeholders, including policy-makers, in the sample, we would have potentially been able to identify additional factors relating to social, cultural and political barriers to the use and uptake of research findings in politics and practice. However, constraints such as access to local stakeholders, language barriers and time zones drove our decision to focus on researchers. A future synthesis effort would need to include the other voices.

## Conclusions

There is ever growing awareness of how critical it is to close the gap between policy-makers, practitioners and researchers. Using the researchers’ perspectives, in this article we give insight into three different strategies that can facilitate this process, with the first strategy requiring proactive searching for the latest findings on the part of well-informed policy-makers, the second requiring researchers to take steps to ensure an active exchange of ideas and information with diverse stakeholders when designing the research project and ensuring the latter’s involvement throughout; and the third using a transdisciplinary and/or co-creation approach to establish equal partnerships and trust among all involved stakeholders.

The five case studies reported here also show some of the difficulties that prevail for research to be taken up into policy and practice, despite everyone’s best intentions and efforts. Researchers may not always be best placed for communication, dissemination and advocacy work, all activities which are very time intensive or become important only towards the end of a research project when clear and high-quality evidence is produced. Moreover, it takes a strong body of evidence, advocacy and coalition building with appropriate stakeholders to influence policy, and then a further major effort of resources to see policy followed through into practice. It is through experiences such as this synthesis initiative that precious insights and learning can be gained for the common good of all involved moving forward, and it is crucial that funders continue to support and/or adapt their funding schemes to ensure some of these strategies are implemented.

## Data Availability

The datasets used and/or analysed during the current study are available from the corresponding author on reasonable request.

## Abbreviations

ART: Antiretroviral therapy
CAG: Country Advisory Group
COHESION: Community Health System Innovation
HIA: Health impact assessment
HIA4SD: Health impact assessment for engaging natural resource extraction projects in sustainable development in producer regions
HIV: Human immunodeficiency virus
IAS: International Aids Society
Jazia PVS: Jazia Prime Vendor System
LMICs: Low- and middle-income countries
MoH: Ministry of Health
MGCSP: Ministry of Gender Children and Social Protection
NADEL: Center for Development and Cooperation at the Swiss Federal Institute of Technology
NHIA: National Health Insurance Authority
NHIS: National Health Insurance Scheme
r4d Programme: Swiss Programme for Research on Global Issues for Development
SDC: Swiss Agency for Development and Cooperation
SNSF: Swiss National Science Foundation
Swiss TPH: Swiss Tropical and Public Health Institute
UHC: Universal health coverage
WHO: World Health Organization
